# Semi-Solid Nutrients for Prevention of Enteral Tube Feeding-Related Complications in Japanese Population: A Systematic Review and Meta-Analysis

**DOI:** 10.3390/nu12061687

**Published:** 2020-06-05

**Authors:** Yoji Kokura, Chieko Suzuki, Hidetaka Wakabayashi, Keisuke Maeda, Kotomi Sakai, Ryo Momosaki

**Affiliations:** 1 Department of Clinical Nutrition, Keiju Medical Center, Ishikawa 926-8605, Japan; 2 Department of Medical Nutrition, Graduate School of Human Life Science, Osaka City University, Osaka 558-8585, Japan; 3 Department of Internal Medicine, Ajisu Kyoritsu Hospital, Yamaguchi 754-1277, Japan; bells-t2@rc4.so-net.ne.jp; 4 Department of Rehabilitation Medicine, Yokohama City University Medical Center, Kanagawa 232-0024, Japan; noventurenoglory@gmail.com; 5 Department of Geriatric Medicine, National Center for Geriatrics and Gerontology, Aichi 474-8511, Japan; kskmaeda1701@gmail.com; 6 Department of Palliative and Supportive Medicine, Graduate School of Medicine, Aichi Medical University, Aichi 480-1195, Japan; 7 Department of Rehabilitation Medicine, Setagaya Memorial Hospital, Tokyo 158-0092, Japan; koto.sakai1227@gmail.com; 8 Department of Rehabilitation Medicine, Mie University Graduate School of Medicine, Mie 5148507, Japan; momosakiryo@gmail.com

**Keywords:** enteral nutrition, tube feeding, gastrointestinal complications, gastroesophageal reflux, pneumonia, diarrhea, constipation

## Abstract

The aim of this systematic review was to assess the best available evidence on semi-solid nutrients for prevention of complications associated with enteral tube feeding (ETF). PubMed (MEDLINE), EMBASE, Cochrane Central Register of Controlled Trial, Ichushi-web, and World Health Organization International Clinical Trials Registry Platform databases were searched for relevant articles. Randomized controlled trials (RCTs), cluster RCTs, and crossover trials comparing the effects of semi-solid nutrients with those of control interventions in patients on ETF were included in the review. The primary outcome was development of gastroesophageal reflux (GER). Eight RCTs and five crossover trials involving 889 study participants in total were examined via meta-analysis. The meta-analysis showed that semi-solid nutrients significantly decreased the risk of GER (risk ratio 0.39; 95% confidence interval (CI) 0.21 to 0.73) and the GER index (mean difference −2.93; 95% CI −5.18 to −0.68). Dwell time in the stomach was significantly shortened (standardized mean difference (SMD) −0.50; 95% CI −0.99 to −0.02), as was care time defined as the time needed to prepare and administer the nutrient solution (SMD −8.02; 95% CI −10.94 to −5.10). Semi-solid nutrients significantly decrease the risk of GER and the dwell time in the stomach in adult patients.

## 1. Introduction

Enteral tube feeding (ETF) plays a major role in the management of patients with poor voluntary food intake, those with chronic neurological or mechanical dysphagia or gut dysfunction, and those who are critically ill [1]. ETF is widely used in acute and subacute care, rehabilitation, long-term care, and home settings [2]. ETF is provided to maintain gut integrity, modulate stress and the systemic immune response, and attenuate disease severity [3,4,5,6]. The logistics of administering ETF may appear less complex than those involved in parenteral nutrition, but serious harm and even death can result from the adverse events that can occur during the process of ordering, administering, and monitoring ETF [7].

The potential adverse events associated with ETF include clinical complications such as gastrointestinal complications [2]. High gastric residuals have been reported to occur in 39% of patients receiving ETF, constipation in 15.7%, diarrhea in 2.0–95.0%, abdominal distention in 12.2%, vomiting in 13.2%, nausea in 10–20%, regurgitation in 0.4–6.0%, and pneumonia in 12.5–30.0% [1,6,8,9,10]. Furthermore, withdrawal of ETF as a consequence of uncontrollable gastrointestinal complications has been reported to occur in 15.2% of patients [8]. These complications prevent completion of ETF in situations where nutritional management is necessary. Therefore, it is crucial to prevent gastrointestinal complications.

It is unclear whether use of semi-solid nutrients is effective for prevention of complications in patients with ETF. Semi-solid nutrients are used as a nutritional management method intended to prevent these complications by increasing the viscosity or changing the shape of the nutrient material injected through the tube. In Japan, semi-solid nutrient pharmaceuticals were launched in 2014. They are inexpensive, covered by insurance, and can be used in a variety of settings. In recent years, semi-solid nutrient formulations with higher viscosity than liquid nutrients have been reported to be effective in reducing gastroesophageal reflux (GER) in patients who have undergone percutaneous endoscopic gastrostomy [11,12,13].

A systematic review of trials examining the efficacy and the acceptability of semi-solid nutrients would be informative and useful for clinicians and researchers. Previous studies have reached inconsistent conclusions due to differing results, study quality, and effect sizes. A systematic review can provide comprehensive evidence through systematic searching, identification, selection, evaluation, and integration.

The aim of this systematic review was to assess the best available evidence on semi-solid nutrients for prevention of complications associated with ETF. We hope the findings will guide future directions of research in this field.

## 2. Materials and Methods

The protocol for this review was prospectively registered with the PROSPERO database for systematic reviews (CRD42018110004).

### 2.1. Types of Studies

Randomized controlled trials (RCT)s, cluster RCTs, and crossover trials were eligible for inclusion in the meta-analysis. Abstracts and non-English language publications were included.

### 2.2. Types of Participants

Studies involving patients aged ≥20 years were eligible for inclusion regardless of sex or indication for ETF.

### 2.3. Types of Interventions and Comparisons

We reviewed studies that included semi-solid nutrient interventions intended to prevent complications associated with ETF. Control interventions were defined as liquid nutrients. The major difference between the two interventions is the dynamic viscosity of the feeds. A semi-solid nutrient is defined as a nutritional management method that entails “increasing the viscosity or changing the shape of a nutrient material injected through a tube” [14].

Semi-solid nutrients can be divided into two types. The first are commercially available semi-solid nutrients. More than 10 of these products are available on the market in Japan, including semi-solid nutrient pharmaceuticals that were first launched in Japan in 2014. Semi-solid formulas and food additives are used to increase the viscosity of liquid formulas by 2000–20,000 mPa·s. The package insert for semi-solid nutrients pharmaceuticals typically states, “The standard dose for adults is 1200–2000 kcal/day administered several times a day in divided doses directly into the stomach via a gastrostomy tube. The administration time is 2–3 min per 100 g (6–9 min per 300 g), and the maximum single dose is 600 g.”

The second type are nutrients whose viscosity has been adjusted by adding a thickener or gelling agent (agar, gelatin, pectin, carrageenan, starch, guar gum, or xanthan gum) to commercially available liquid nutrients. Semi-solid nutrients can be directly administered via a feeding tube or nutrients, and the thickener or gelling agent can be separately administered through the feeding tube such that partial solidification occurs in the stomach.

### 2.4. Outcomes

The primary outcome was development of GER. Secondary outcomes were rates of pneumonia, diarrhea, constipation, pressure ulcer, leak from the gastrostomy tube, dwell time in the stomach, care time (time taken to prepare and administer the nutrient solution), rehabilitation time, activities of daily living, and medical costs.

### 2.5. Search Strategy

**Figure A1:**
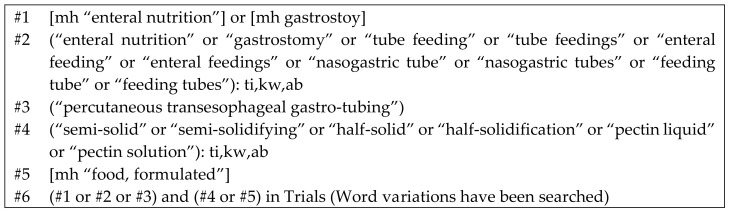
CENTRAL search strategy.

**Figure A2:**
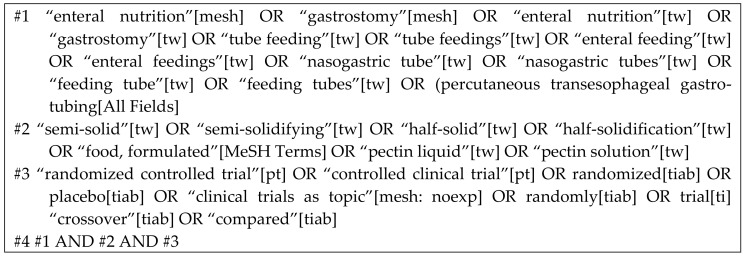
MEDLINE search strategy (PubMed).

**Figure A3:**
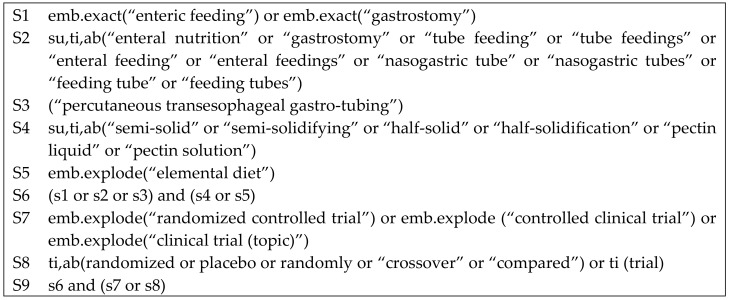
EMBASE search strategy (Pro Quest).

**Figure A4:**
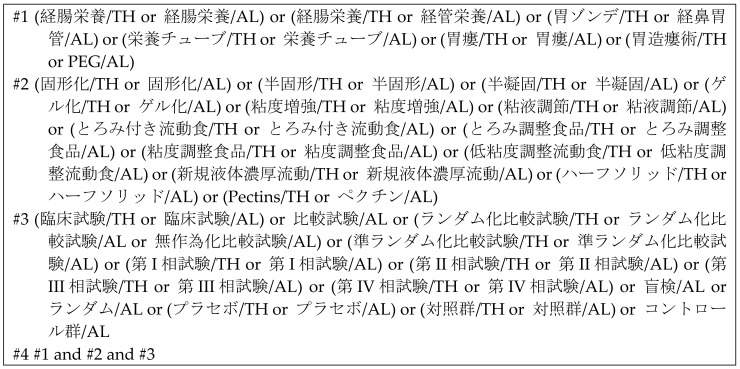
Ichushi-web search strategy.

**Figure A5:**
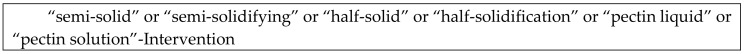
WHO-ICTRP search strategy.

All relevant published studies were identified by searching the following databases: PubMed (MEDLINE), Cochrane Central Register of Controlled Trials (CENTRAL), EMBASE, and Ichushi-web, which is a Japanese journal database. All searches were performed for publications from the inception of each database until July 2019 (Figure A1, Figure A2, Figure A3 and Figure A4). We also searched for ongoing studies using the World Health Organization International Clinical Trials Registry Platform (ICTRP) (www.who.int/ictrp) (Figure A5).

### 2.6. Selection of Studies

Two authors (Y.K., C.S.) independently reviewed all potentially eligible studies by examining the title and the abstract and, where necessary, the full-text version of the paper. If agreement could not be reached by discussion, a third author (R.M.) made the final decision about eligibility.

### 2.7. Data Extraction and Management

Two authors (Y.K., C.S.) worked independently and used a standardized form to extract study characteristics and outcome data from the included studies. The original data were checked if a discrepancy was found, and any disagreements were resolved by a third author (R.M.).

### 2.8. Risk of Bias Assessment

The methodological quality of the selected studies was assessed as recommended by the Cochrane Review Groups [15]. The same two authors (Y.K., C.S.) independently performed the quality assessment. Any disagreements between authors was resolved by discussion. We contacted the authors of the primary studies in the event of missing data. A risk of bias table was created that included a description and judgment (low risk, high risk, or unclear risk) of the following seven domains for each of the included studies: (1) random sequence generation; (2) allocation concealment; (3) blinding of participants and personnel; (4) blinding of outcome assessment; (5) incomplete outcome data; (6) selective reporting; and (7) other sources of bias.

### 2.9. Statistical Analysis

For all outcomes related to continuous data, we calculated a pooled estimate of the standardized mean difference (SMD) with a 95% confidence interval (CI). The mean difference (MD) was used for continuous data if the outcomes were measured in the same way between trials. Dichotomous data are presented as the summary risk ratio (RR) with a 95% CI. We used fixed-effect meta-analysis of the combined data where it was reasonable to assume that the studies were estimating the same underlying treatment effect. We used the I2 statistic to assess heterogeneity. An I2 of 50% was considered to reflect substantial heterogeneity. If the I2 was more than 50%, we used random-effects analysis to combine the data. The threshold for significance was set at *p* = 0.05. The EZR package was used for all statistical analyses [16].

## 3. Results

The protocol for this review was prospectively registered with the PROSPERO database for systematic reviews (CRD42018110004).

### 3.1. Study Selection

**Figure 1:**
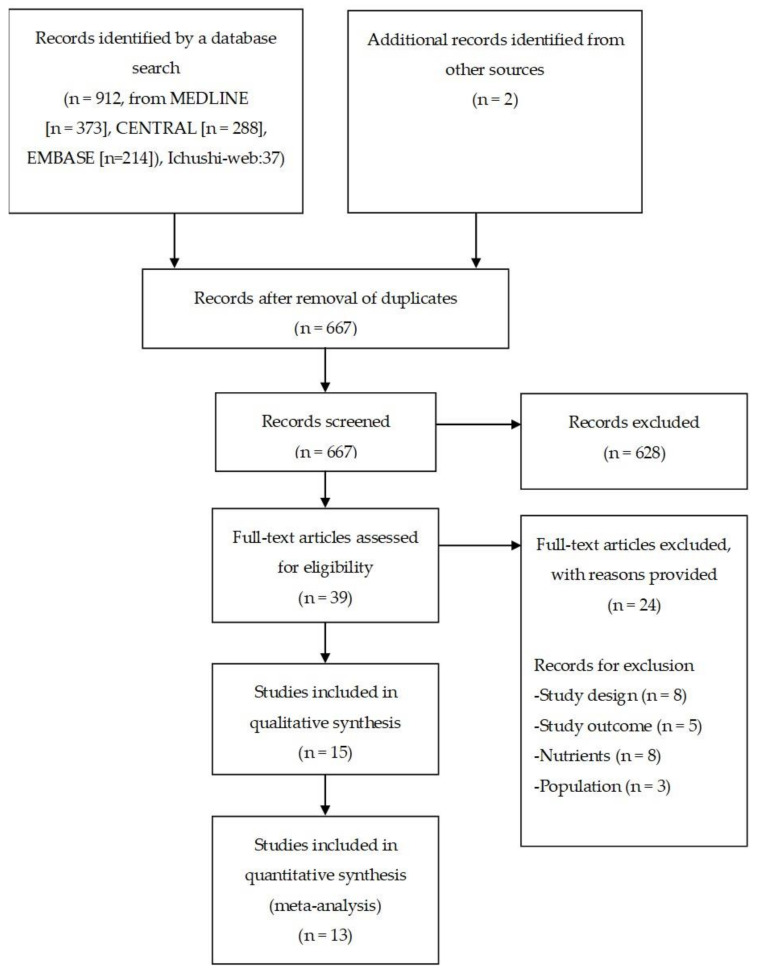
Flowchart of the study selection procedure.

After screening 912 records, 39 potentially relevant studies were identified (Figure 1). Fifteen of these 39 studies met the study inclusion criteria (Muramatsu et al., 2018 [17]; Ishii et al., 2006 [18]; Togashi & Paku 2012 [19]; Paku et al., 2012 [20]; Nakahori et al., 2011 [21]; Abe et al., 2011 [22]; Muramatsu et al., 2010 [23]; Kanie et al., 2004 [11]; Shizuku et al., 2011 [24]; Nagasawa 2009 [25]; Toh et al., 2016 [26]; Tabei et al., 2018 [27]; Nishiwaki et al., 2009 [12]; Shimizu et al., 2016 [13]; Higashiguchi et al., 2014 [28]). The 15 studies comprised 10 RCTs [17,18,19,20,21,22,23,26,27,28] and five crossover trials [11,12,13,24,25]. Six studies [18,19,20,21,22,23] were published in abstract form only. The details of each study are shown in Table 1. The first study was published in 2004, and the most recent was published in 2019. All studies were published in English or Japanese. Two ongoing studies were identified: “The effects of PEG tube feeding of semi-solid nutrients on salivation” (JPRN-UMIN000006732] and “A more physiological feeding process in ICU: the intermittent infusion with semi-solidification of nutrients (ClinicalTrials.gov NCT03017079)”.

### 3.2. Patient Characteristics

Table 1 provides a comprehensive summary of the 15 studies. Mean age of participants ranged from 76.2 to 85.8 years, and 53.3–76.5% were women. Five studies involved inpatients with pre-existing gastrostomy, and 11 involved patients with a new percutaneous endoscopic gastrostomy. ETF was administered via a gastrostomy tube in 15 studies; in one of these studies, ETF was delivered via a nasogastric tube in some patients [12]. In these 15 studies, 33.3–100% of patients had stroke [11,12,13,17,24,25,26,27,28], 20.0–60.0% had dementia [11,12,17,25,26,27], 46.1% had respiratory disorders [26], 18.5% had dysphagia [17], 15.3% had neurodegenerative disorders [26], 13.7% had malignancy [26], 6.7% had neuromuscular disorders [12], 7.4% had hypoxic encephalopathy [27], 7.4% had esophageal cancer [27], 3.7% had gastric cancer [27], 3.7% had brain tumor surgery [27], 3.7% had cerebral contusion [27], and 3.7% had disuse syndrome [27]. Comorbidities were as follows: 12.1–25.6% had diabetes mellitus [13,26], 3.4–86.4% had hiatal hernia [12,13,26], 40% had reflux esophagitis [12], 10.6% had pneumoperitoneum [13], 4.5% had gastric bleeding [13], and 2.6% had partial gastric resection [26]. In one trial [25], 70% of patients were taking a proton-pump inhibitor.

### 3.3. Intervention

The viscosity of the semi-solid nutrient was increased by Easy Gel^®^ (Otsuka, Tokyo, Japan) [13,17,25], agarose [11,12,28], MEDI-F Pushcare^®^ (Nestle, Kobe, Japan) [24], PG Soft^®^ (Terumo, Tokyo, Japan) [26], or REF-P1^®^ (Nutri, Mie, Japan) [27]. The viscosity (mPa·s, cP) of the semi-solid nutrient in each study was 1000 [27], 6000 [13], 6500–12,500 [28], or 20,000 [17,19,20,24,26]. The amount of energy administered was 300–400 kcal/feed [27], 450 kcal/feed [25], or 600 kcal/feed [28] with an upper limit of 1200 kcal [19,20]. The semi-solid nutrient was administered as a bolus over 5–10 min [26], 10 min [17], 15 min [19,20], or 10–20 min [28]. The observation period was 1–28 days.

### 3.4. Outcomes

Of the four trials that assessed GER [11,12,13,18], three categorized it dichotomously as present or absent [11,13,18], and the remaining trial used the GER index [12]. Intragastric and esophageal distribution were monitored using a scintillation camera. Reflux of contrast agent into the esophagus was observed on radiographic examination. Thirty minutes after administration of the contrast agent, a computed tomography scan of the esophagus was performed with a slice thickness of 1 cm. GER was confirmed if the Hounsfield number exceeded 100 in each examined slice [11]. The upper gastrointestinal tract was observed radiologically from onset to 1 min after the end of administration of the contrast agent. GER was considered present if any reflux of contrast agent into the esophagus was observed [13]. Intragastric and esophageal distribution were monitored using a scintillation camera in the supine position. The radioactivity of the esophagus and the stomach was determined at a rate of one frame every 150 s using a scintillation camera for up to 90 min after bolus infusion of 200 mL radiolabeled liquid or semi-solid nutrients through percutaneous endoscopic gastrostomy. The GER index was defined as the maximal percentage of esophageal radioactivity count to total infused radioactivity [12].

Pneumonia, diarrhea, constipation, and leak from the gastrostomy site were also categorized dichotomously (present or absent). Pneumonia was diagnosed based on the following: the Japanese Respiratory Society guidelines [17]; clinical symptoms confirmed by radiologic findings or detection of enteral feed material in aspirate from the trachea26; body temperature ≥37.5 °C; respiratory symptoms; abnormal blood test results, including for white blood cell count and C-reactive protein level; and infiltrative shadow observed on chest radiography or chest computed tomography [27].

Diarrhea was defined as watery or muddy stools [18,24], watery or muddy stools more than three times a day [21]; a King’s Stool Chart score of ≥15 [26], or watery or soft stools more than five times a day [27]. Dwell time in the stomach was assessed by Tlag (time to peak excretion) [25], T1/2 (half-emptying time) [25], and gastric emptying time determined as the time required for 50% of the initial radioactivity to empty from the stomach [12]. Care time included the amount of time needed to prepare and administer the nutrients and water [22,24,27,28].

### 3.5. Risk of Bias Assessment

**Figure 2:**
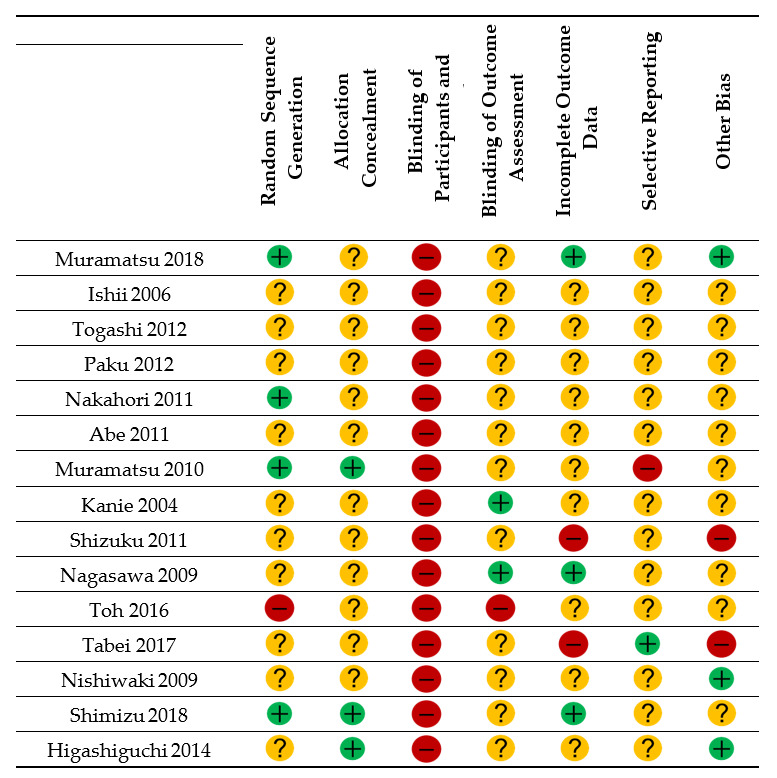
Risk of bias summary. 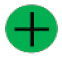 = low risk of bias; 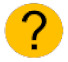 = unclear; 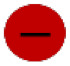 = high risk of bias.

Four of the 15 studies performed random sequence generation appropriately [13,17,21,23] (Figure 2). Nine studies did not report the methods used for random sequence generation and were classified as unclear [11,12,18,19,20,22,24,25,27,28]. Twelve studies did not report allocation concealment [11,12,17,18,19,20,21,22,24,25,26,27]. None of the studies performed blinding of participants and observers, and all were judged to have a high risk of bias [11,12,13,17,18,19,20,21,22,23,24,25,26,27,28]. Two of the studies [11,25] included appropriate blinding for outcome assessment. Three of the 15 studies were at low risk of bias for incomplete outcome data [13,17,25]. Only one study [27] did not have selective reporting and was judged to have a low risk of bias. Two studies contained other types of bias and were deemed to have a high risk. One study [27] scored low for required sample size. In one study [24], only half the study period specified in the study protocol was completed, thereby shortening the observation period. Furthermore, we could not obtain missing outcome data for one study [22] and could not perform a quantitative synthesis for another [23], leaving data for 13 studies available for quantitative analysis (Table 2).

### 3.6. Quantitative Synthesis

#### 3.6.1. Gastroesophageal Reflux

Four trials [11,12,13,18] (including 210 participants) included GER data. The meta-analysis showed that ETF administered in the form of semi-solid nutrients significantly decreased the prevalence of GER (RR 0.39; 95% CI 0.21–0.73; *p* = 0.003; I2 = 0%; Table 2). One trial [12] (with 30 participants) reported that semi-solid nutrients significantly decreased the GER index (MD −2.93; 95% CI −5.18, −0.68; *p* = 0.011).

#### 3.6.2. Pneumonia

Seven trials [17,19,20,21,26,27,28] (with 615 participants) assessed pneumonia. We could not find any significant effect of ETF using semi-solid nutrients on pneumonia (RR 0.99; 95% CI 0.51, 1.93; *p* = 0.970; I2 = 58.0%).

#### 3.6.3. Diarrhea

Eight trials [18,19,20,21,24,26,27,28] (including 541 participants) reported data on diarrhea. Meta-analysis showed that ETF with semi-solid nutrients had no significant effect on diarrhea (RR 0.82; 95% CI 0.57–1.18; *p* = 0.287; I2 = 47.4%).

#### 3.6.4. Constipation

Only one trial [28] (112 participants) reported data on the incidence of constipation. We found that ETF with semi-solid nutrients did not significantly reduce the risk of constipation (RR 0.25; 95% CI 0.03–2.17; *p* = 0.208).

#### 3.6.5. Leak from Gastrostomy Site

Only one trial [18] (with 14 participants) reported data on the incidence of leak from the gastrostomy site. Meta-analysis showed that ETF with semi-solid nutrients did not significantly decrease the risk of leak from the gastrostomy site (RR 0.20; 95% CI 0.01–3.50; *p* = 0.271).

#### 3.6.6. Dwell Time in the Stomach

Two trials [12,25] (including 70 participants) reported data on dwell time in the stomach. Meta-analysis showed that ETF administered in the form of semi-solid nutrients significantly shortened the dwell time (SMD −0.50; 95% CI −0.99, 0.02; *p* = 0.043; I2 = 50%).

#### 3.6.7. Care Time

Three trials [24,27,28] (including 369 participants) reported data on care time. Meta-analysis showed that semi-solid nutrients significantly shortened care time (SMD −8.02; 95% CI −10.94, −5.10; *p* < 0.001; I2 = 95.2%).

#### 3.6.8. Pressure Ulcer, Rehabilitation Time, Activities of Daily Living, and Medical Costs

None of the studies reported on pressure ulcer, rehabilitation time, activities of daily living, or medical costs.

## 4. Discussion

### 4.1. Summary of Results

Our literature search identified 15 eligible randomized trials with a total of 946 participants. Meta-analysis was possible for GER, pneumonia, diarrhea, constipation, leak from the gastrostomy site, and dwell time in the stomach. We found that administration of semi-solid nutrients significantly decreased the risk of GER, dwell time in the stomach, and care time but had no statistically significant effect on rates of pneumonia, diarrhea, constipation, or leak from the gastrostomy site. However, all outcomes were measured in a small number of trials. Therefore, our findings should be interpreted with caution because the quality of the evidence was often unclear and differed among the outcome measures.

### 4.2. Overall Completeness and Applicability of Evidence

The results of the review are limited by a number of factors. First, six of the studies were published in only abstract form, which explains why many risks of bias were evaluated as unclear. Second, all patients included in the meta-analysis were Japanese, which limits the generalizability of the data to other populations. Therefore, in the future, research targeting non-Japanese participants should be conducted. Third, there may have been differences in the types of patients included in the studies. Known indications for ETF include stroke and dementia, and almost all the patients had one of these conditions; however, comorbidities were less clear. Furthermore, the effects of medication could not be investigated. Fourth, the patients either already had an established gastrostomy or had a new percutaneous endoscopic gastrostomy, thus the backgrounds of the patients were not exactly the same. Fifth, in the trials that evaluated semi-solid nutrients, none of the products were identical in composition, formulation, or quantity. Stronger evidence would be obtained by performing trials with the same semi-solid nutrients. Sixth, the definition of complications was inconsistent and was unclear in some studies. We contacted the authors by post and e-mail to clarify definitions used but three corresponding authors could not be reached. The exact definition of complications should be standardized for future trials to obtain high-quality results. Seventh, the longest follow-up period was 28 days. Therefore, the long-term effects of this intervention remain unclear. Finally, the meta-analysis for GER as a primary outcome was performed for only four trials with a total of only 210 participants. Therefore, it may be premature to perform meta-analysis of the data for GER at this stage. However, it is important to consider the best available evidence in order to indicate the directions of future research.

### 4.3. Quality of Evidence

In general, the quality of reporting was poor. Most trials reported random assignment of patients, but the methods of randomization were not described in full detail. Although all trials used semi-solid nutrients in the intervention group, the success of blinding was not recorded. Studies of semi-solid nutrients are difficult to perform with blinding of participants and personnel because they have higher viscosity and are clearly different in appearance from liquid nutrients. There was large heterogeneity between the studies, particularly for care time. The potential problem associated with this heterogeneity relates to the definition of care time. The definition of care time was variously “infusion time” [27,28] or “time for preparing nutrients and for administering nutrients and water” [24] and differed from study to study.

### 4.4. Potential Biases in the Review Process

We were unable to assess potential reporting bias because of the small number of studies in the review, which prevented us from constructing a funnel plot. Second, the included patients were adults aged ≥20 years and all had received ETF in a hospital setting. Therefore, our results cannot be applied to younger patients or those using ETF at home. Finally, a potential source of bias of this review may originate from the search strategy, which detected only English and Japanese language publications.

### 4.5. Agreements and Disagreements with Other Studies or Reviews

To our knowledge, no review has previously examined the impact of semi-solid nutrients on complications of ETF.

## 5. Conclusions

This systematic review and meta-analysis found that semi-solid nutrients significantly decreased the risk of GER and also decreased the dwell time in the stomach and the care time for ETF in adult patients. However, use of semi-solid nutrients did not decrease the rates of pneumonia, diarrhea and constipation, or leak from the gastrostomy site. The limitations of this study were the small number of trials included and the lack of high-quality evidence. Large-scale, high-quality, prospective randomized studies are needed to further investigate the impact of semi-solid nutrients on complications in patients receiving ETF. Currently, some clinical trials are under way (JPRN-UMIN000006732, ClinicalTrials.gov. NCT03017079) and further evidence is awaited.

## Figures and Tables

**Table 1:** Characteristics of included studies.

Study	Country	Participants	n (I/C)	Intervention	Control	Outcomes	Conclusion
Muramatsu 2018	Japan	Patients who agreed to study participation before gastrostomy	151 (75/76)	Semi-solid enteral nutrients prepared by adding pectin and calcium with a viscosity of 20,000 cP	Liquid enteral nutrients	Pneumonia	Semi-solid enteral nutrients decreased the risk of pneumonia
Ishii 2006	Japan	Inpatients with gastrostomy	14 (7/7)	Semi-solid enteral nutrients	Liquid enteral nutrients	GER ^1^ Diarrhea Leak from the gastrostomy site	Diarrhea and leak from the gastrostomy site were less common with semi-solid nutrients than with liquid enteral nutrients
Togashi 2012	Japan	Inpatients with gastrostomy	94 (50/44)	Semi-solid enteral nutrients with viscosity of 20,000 cP	Liquid enteral nutrients	Pneumonia Diarrhea	Patients on semi-solid enteral nutrients had a lower incidence of pneumonia and diarrhea
Paku 2012	Japan	Inpatients with gastrostomy	94 (50/44)	Semi-solid enteral nutrients with viscosity of 20,000 cP	Liquid enteral nutrients	Pneumonia Diarrhea	Patients on semi-solid enteral nutrients had a lower incidence of pneumonia and diarrhea
Nakahori 2011	Japan	Inpatients with gastrostomy	20 (10/10)	Semi-solid enteral nutrients	Liquid enteral nutrients	Pneumonia Diarrhea	Pneumonia and diarrhea were difficult to evaluate because of the small number of cases
Abe 2011	Japan	Inpatients with gastrostomy	15 (8/7)	Semi-solid enteral nutrients	Liquid enteral nutrients	Diarrhea Care time	Semi-solid enteral nutrients were associated with decreased occurrence of diarrhea and shorter care time
Muramatsu 2010	Japan	Inpatients with PEG ^2^	22 (11/11)	Semi-solid enteral nutrients	Liquid enteral nutrients	Consistency of stools	Consistency of stools improved from watery to solid in the semi-solid nutrient group
Kanie 2004	Japan	Patients being fed by PEG	34 (17/17)	Half-solid enteral nutrients were prepared by mixing with 5 g of agarose	Liquid enteral nutrients	GER	The rate of GER was lower in the half-solid nutrient group
Shizuku 2011	Japan	Elderly patients undergoing PEG feeding	64 (32/32)	Half-solid enteral nutrients MEDI-F Pushcare^®^ (Nestle, Kobe) with viscosity of about 2000 mPa·s	Liquid enteral nutrients	Diarrhea Care time needed	The care time needed was significantly less in the half-solid enteral nutrient group; the numbers of patients who developed diarrhea were similar between the groups
Nagasawa 2009	Japan	Patients more than 1 week after gastrostomy	20 (10/10)	Semi-solid nutrients prepared by adding Easy gel to RACOL^®^ (Otsuka, Tokyo)	Liquid enteral nutrients (RACOL^®^, Otsuka, Tokyo)	Dwell time in the stomach	Semi-solid enteral nutrients accelerate gastric emptying during the early phase when compared with liquid enteral nutrients
Toh 2016	Japan	Patients who received gastrostomy for enteral nutrition Tube feeding for 2 weeks prior to gastrostomy	117 (45/72)	Semi-solid enteral feed with a dynamic viscosity of 20,000 cP	Liquid feed with dynamic viscosity of 5–10 mPa s	Pneumonia Diarrhea	Using semi-solid enteral feeds may reduce the risk of pneumonia No statistically significant difference in the rates of diarrhea between the two groups
Tabei 2018	Japan	Age ≥20 years Patients who needed nutritional therapy via a percutaneous endoscopic gastrostomy, percutaneous transesophageal gastric tube, or a nasogastric tube	27 (15/12)	Pectin solution with viscosity of 1000–2000 mPa·s	Liquid enteral nutrition diet of K-LEC^®^ (Kewpie Corporation, Tokyo) with viscosity of 5 mPa·s	Pneumonia Diarrhea Care time	No cases of pneumonia in either group No between-group difference in incidence of diarrhea Pectin solution was able to be administered in a significantly shorter time than the liquid enteral nutrition diet
Nishiwaki 2009	Japan	Patients more than 1 month after gastrostomy	30 (15/15)	Semi-solid enteral nutrients prepared by adding agar to RACOL^®^ (Otsuka, Tokyo)	Liquid enteral nutrients (RACOL^®^ (Otsuka, Tokyo)	GER Dwell time in the stomach	GER was significantly inhibited by semi-solid enteral nutrients No between-group difference in gastric emptying time
Shimizu 2016	Japan	Patients who planned to undergo PEG for the first time	132 (66/66)	Semi-solid contrast agent with viscosity of 6000 mPa·s	Liquid contrast agent (3 mPa·s)	GER	Semi-solid contrast agents reduced the incidence of GER after PEG
Higashiguchi 2014	Japan	Aged >20 years Patients undergoing PEG or had plans for PEG	112 (56/56)	Semi-solid enteral nutrients with viscosity of 6500–12,500 mPa·s prepared using alginic acid and agar powder	Liquid enteral nutrients with viscosity of 5.51–6.52 mPa·s	Pneumonia Diarrhea Constipation Care time	Semi-solid enteral nutrients were able to be administered in a significantly shorter time than liquid enteral nutrition No statistically significant difference in the rates of pneumonia, diarrhea, and constipation between the two groups

^1^ GER, gastroesophageal reflux; ^2^ PEG, percutaneous endoscopic gastrostomy.

**Table 2:** Summary of meta-analysis for outcomes.

Outcome	Studies, n	Participants (Intervention/Control), n	Effect Size	95% CI ^1^	Inconsistency, I^2^ (%)
Gastroesophageal reflux (present or absent)	3	180 (90/90)	RR: 0.39	(0.21, 0.73)	0
Gastroesophageal reflux (GER ^2^ index)	1	30 (15/15)	MD ^3^: −2.93	(−5.18, −0.68)	-
Pneumonia	7	615 (328/287)	RR: 0.99	(0.51, 1.93)	58.0
Diarrhea	8	541 (292/249)	RR: 0.82	(0.57, 1.18)	47.4
Constipation	1	112 (56/56)	RR: 0.25	(0.03, 2.17)	-
Leak from gastrostomy site	1	14 (7/7)	^5^ RR: 0.20	(0.01, 3.50)	-
Dwell time in the stomach	2	70 (35/35)	^4^ SMD: −0.50	(−0.99, −0.02)	50.0
Care time	3	369 (189/180)	SMD: −8.02	(−10.94, −5.10)	95.2

^1^ CI, confidence interval; ^2^ GER, gastroesophageal reflux; ^3^ MD, mean difference; ^4^ SMD, standardized mean difference; ^5^ RR, risk ratio.
